# Supplementary figures and images for: PhyloString: A web server designed to identify, visualize, and evaluate functional relationships between orthologous protein groups across different phylogenetic lineages
Source: PLoS One. 2024 Jan 26;19(1):e0297010. doi: 10.1371/journal.pone.0297010 (PMC10817156; doi:10.1371/journal.pone.0297010)

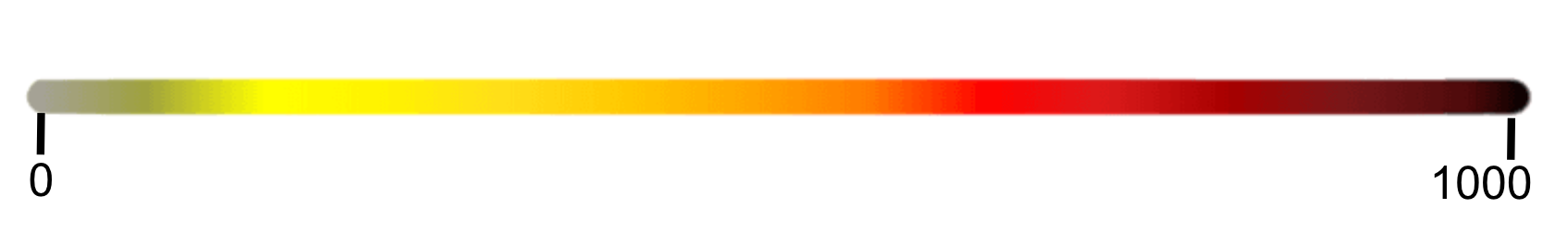

Supplement: S1 Fig — The colors ranged from lighter shades like yellow to darker hues like cherry or black, representing weak to strong functional associations. (TIF) [file pone.0297010.s001.tif]

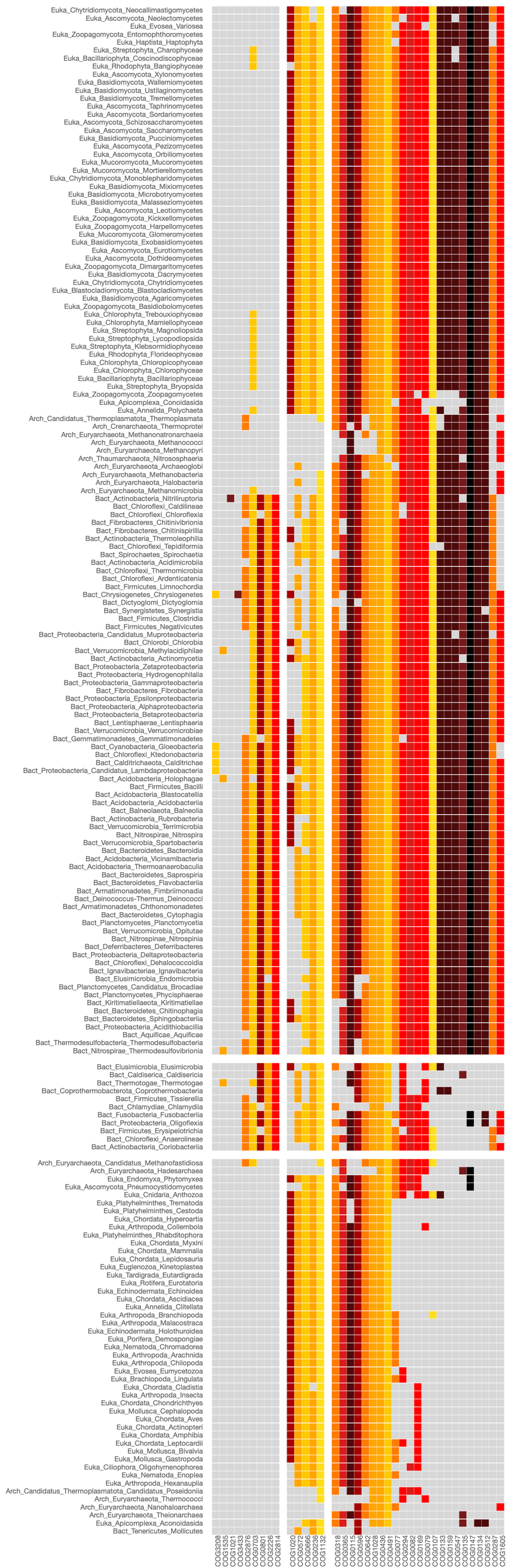

Supplement: S2 Fig — The parameters used in the analysis were: Phylogenetic level: Class; STRING cut-off: 800; COG centers: 3; Phylogenetic centers: 3. The heatmap reveals that certain phylogenetic classes, even though they are part of a phylum typically associated with prototrophic organisms, do not possess the primary enzymes of the L-trp biosynthesis pathway. Examples include the classes Tissierellia, Erysipelotrichia, and Oligoflexia. (TIF) [file pone.0297010.s002.tif]

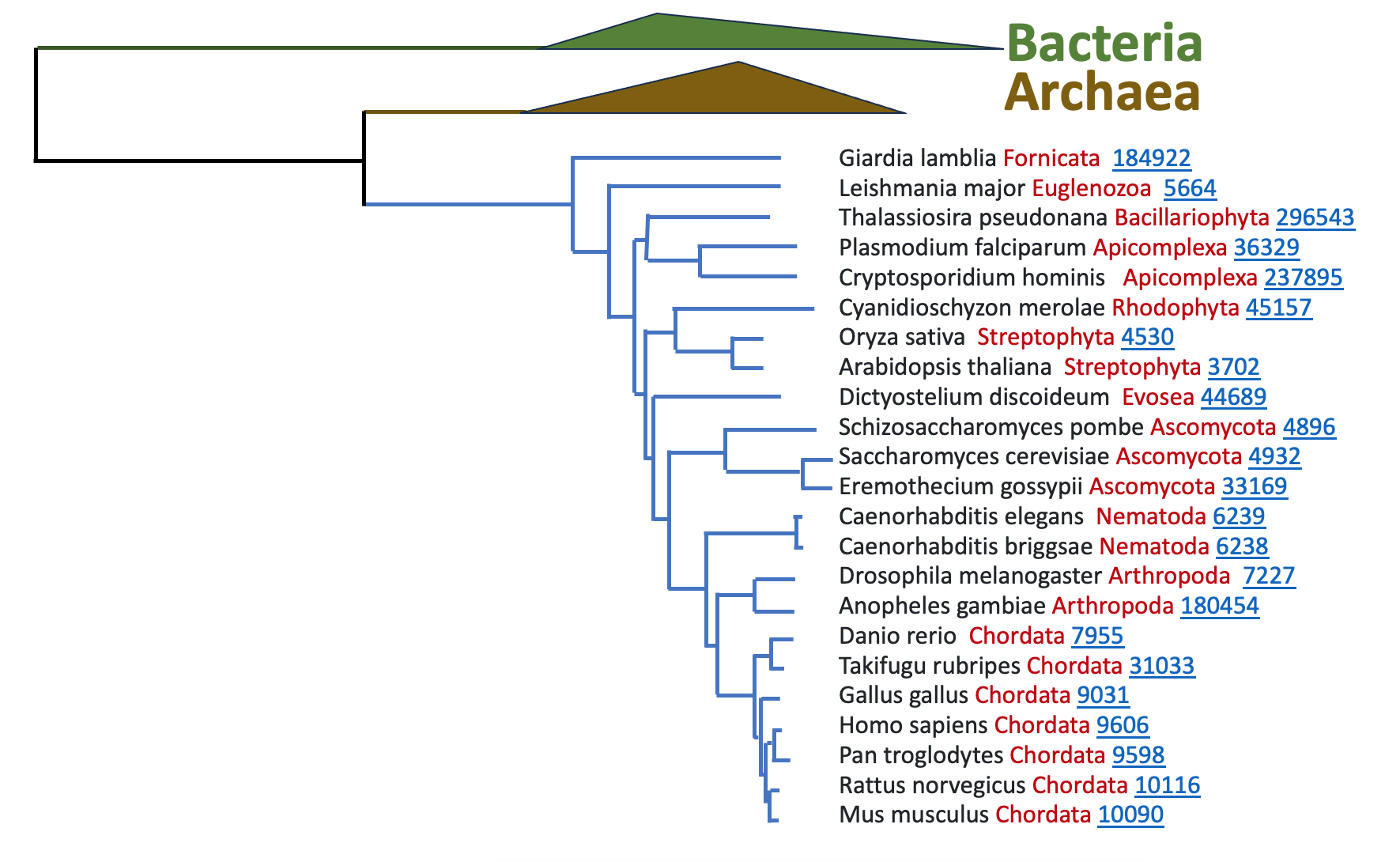

Supplement: S3 Fig — The tree was drawn considering the phylogenetic distances reported for the iterative Tree of Life (iTOL) web page (https://itol.embl.de/). The name, phyla, and Tax ID of representative organisms are listed in black, red, and blue, respectively. (TIF) [file pone.0297010.s003.tif]
